# A Toxicological Assessment of Airborne Microplastics in Beijing

**DOI:** 10.3390/toxics14040312

**Published:** 2026-04-07

**Authors:** Susu Fan, Ziyu Guo, Longyi Shao, Pengju Liu, Tim Jones, Yaxin Cao, Wen-Jing Deng, Hong Li, Kelly BéruBé

**Affiliations:** 1 State Key Laboratory of Coal Resources and Safe Mining, School of Geoscience and Survey Engineering, China University of Mining and Technology, Beijing 100083, China; 2 State Key Laboratory of Regional Environment and Sustainability, School of Environment, Tsinghua University, Beijing 100084, China; 3 School of Earth and Ocean Sciences, Cardiff University, Main Building, Park Place, Cardiff CF10 3AT, UK; 4 Department of Science and Environmental Studies, The Education University of Hong Kong, Tai Po, N.T., Hong Kong, China; wdeng@eduhk.hk; 5 State Key Laboratory of Environmental Criteria and Risk Assessment, Chinese Research Academy of Environmental Sciences, Beijing 100012, China; 6 School of Biosciences, Cardiff University, The Sir Martin Evans Building, Museum Avenue, Cardiff CF10 3AX, UK

**Keywords:** airborne microplastics, dustfall, plasmid scission assay, environmental toxicology, pollution load index

## Abstract

Microplastics have emerged as a relatively new type of pollutant and have attracted significant global attention. This study focuses on toxicology of microplastics in ambient PM_2.5_ and road dustfall in Beijing. It utilizes the Plasmid Scission Assay to toxicologically evaluate the oxidative damage capacity of microplastics as a component of PM_2.5_. The Pollution Load Index (PLI) method, based on the mass concentration of microplastics in ambient air, was employed to assess the ecological risk of atmospheric dustfall microplastics in Beijing. The results showed that both standard microplastic samples and mixed samples of microplastics with ambient PM_2.5_ exhibited a dose–response relationship in DNA damage rates. At the same dose, microplastic samples with smaller particle sizes have a higher DNA damage rate. Based on the PLI results, most road dustfall microplastics in Beijing exhibit significant spatial variation. Analysis of road dustfall along the east–west main road across Beijing’s urban area revealed that microplastic pollution levels are higher in the eastern zone than in the western zone. Comparisons of pollution levels across functional areas in Beijing showed that university areas > residential areas > industrial areas > commercial areas > agricultural areas. In vertically collected samples, higher elevations (PLI_13.6m_ = 3.54) exhibit greater pollution levels than lower (PLI_1.5m_ = 1), which warrants special attention. These findings highlight the complex relationship between atmospheric microplastic accumulation and their oxidative capacity, providing essential insights for the design of targeted emission reduction strategies.

## 1. Introduction

Against the backdrop of rapid industrialization, the extensive application and improper disposal of plastic products have led to increasingly severe plastic pollution, which has become a global environmental challenge [1,2]. As a relatively new type of persistent pollutant, microplastics (MPs) have progressively become a research subject in environmental science, toxicology, and public health since their concept was formally proposed in 2004 [3,4]. This can be attributed to their wide distribution, potentially high toxicity, and significant health risks. Typically, microplastics refers to plastic particles smaller than 5 mm in size [5], which are mainly classified into primary microplastics and secondary microplastics [6]. Primary microplastics are tiny plastic particles directly produced artificially, commonly found in products such as cosmetics and detergents [7]. Secondary microplastics are fragments generated from large plastic products through physical abrasion, chemical degradation, and biodegradation [8]. The atmosphere serves as an important pathway for the regional and global transport of numerous suspended substances including microplastics [9]. Studies have shown that microplastics can be transported via the atmosphere to remote regions such as the Arctic and the Alps [10,11,12,13].

Compared with microplastic pollution in water and soil, atmospheric microplastics exhibit significantly higher mobility and diffusivity [14]. Existing studies have confirmed that atmospheric microplastics can be detected from densely populated urban areas to remote polar and alpine regions [15,16]. Urban areas are characterized by dense population, intensive human activities and heavy traffic, making them highly prone to atmospheric microplastic pollution [17]. As the capital city of China, Beijing is a megacity with a population exceeding ten million, a high level of industrialization, a dense transportation network, and frequent construction activities. Intense human activities may be important sources of atmospheric microplastics [17], including traffic exhaust emissions, construction operations, waste accumulation, and improper disposal of plastic products. Furthermore, PM_2.5_ is a major air pollutant in Beijing. Its particle size range overlaps with the size range of airborne microplastics. The two pollutants often form mixed atmospheric systems. This further increases the complexity of related environmental risks.

The environmental risks presented by microplastics are closely related to their physicochemical properties. Microplastics are persistent pollutants that resist natural degradation. They also have strong adsorption ability and can act as carriers for the transport of toxic substances in the atmosphere, including heavy metals [18], persistent organic pollutants (POPs) [19], and polycyclic aromatic hydrocarbons (PAHs) [20]. There is evidence that shows that microplastics are becoming more mobile, persistent and hazardous in extreme weather, establishing a link with climate change [21]. This increases the risks they present to the environment. Airborne particulate plastics pose a significant health risk due to inhalation and subsequent deposition in the human body, potentially leading to adverse effects. These particulate plastics, originating from various sources such as roadside dust and plastic debris, can contaminate the atmosphere and be inhaled over long distances, affecting human health [22,23]. In addition, atmospheric microplastics can enter the human respiratory tract through inhalation [24]. Nanoscale microplastics can even cross the alveolar barrier into the bloodstream, causing damage to human organs and cells [25].

At present, there is a paucity of domestic and international research on atmospheric microplastics. Most studies focus on the investigation of pollution characteristics, emphasizing the analysis of basic information such as particle size distribution, abundance and composition of microplastics [26,27], whereas research on their toxicological effects is relatively sparce [28]. Studies on the combined toxicity of microplastics in a mixed state with other atmospheric pollutants (e.g., PM_2.5_) are still scarce.

Plasmid Scission Assay (PSA) is an in vitro method used to evaluate the degree of oxidative damage to DNA caused by particulate matter [29]. It indirectly reflects the structural damage of DNA caused by particulate matter by measuring the proportion of different forms (supercoiled, relaxed, linear) of DNA in gel electrophoresis [30]. This method uses the principle of agarose gel electrophoresis to analyze the difference in migration speed of different morphologies of DNA. Supercoiled DNA with intact structure moves the fastest in the electrophoresis instrument, while relaxed DNA moves the slowest. PSA has been widely used to evaluate the genetic toxicity and health effects of different particulate matter samples, such as urban air and dust particles [31,32]. By analyzing the changes in the proportion of relaxed DNA and linear DNA, the oxidative damage level of the sample can be evaluated semi-quantitatively, and then the degree of DNA damage caused by particulate matter can be inferred. PSA is of great significance in assessing the genotoxicity of particulate matter and provides an effective means to study the impact of particulate matter on human health. In recent years, the Pollution Load Index (PLI), as a simple and efficient assessment method, has been widely applied to the risk assessment of pollutants in media such as soil and water [33,34,35,36]. However, studies applying it to the ecological risk assessment of microplastics in atmospheric dustfall are relatively limited. Further research is needed to improve the ecological risk assessment system for atmospheric microplastics.

Focusing on microplastic pollution in the atmospheric environment of Beijing, this study took atmospheric PM_2.5_ and atmospheric dustfall as the research subjects. It aims to systematically investigate the pollution distribution patterns, toxicity effects, and potential ecological risks of atmospheric microplastics in Beijing, thereby providing scientific evidence and data support for regional atmospheric microplastic pollution control and ecological safety assurance. The PSA method was used to evaluate the oxidative damage potential of microplastics as a component of PM_2.5_. In addition, the PLI method, combined with the abundance of microplastics in atmospheric dustfall, was adopted to comprehensively assess the ecological risks of microplastics in atmospheric dustfall in Beijing. It also aimed to clarify the differences in pollution characteristics and ecological risks of atmospheric microplastics along Chang’an Avenue, across different functional zones and at various vertical heights in Beijing.

## 2. Materials and Methods

### 2.1. Sample Collection

**Figure 1:**
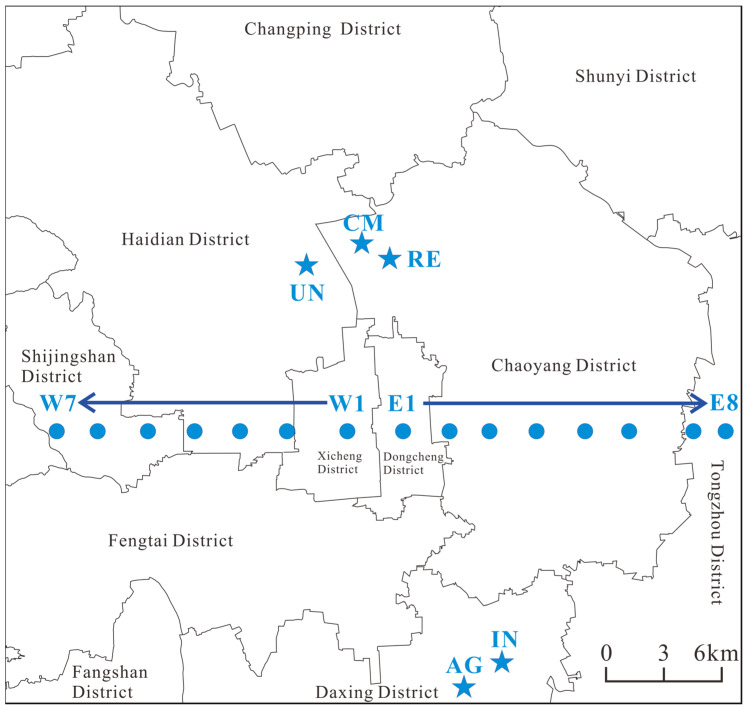
Location of the study area and distribution of sampling sites. Sampling sites from west to east: W7–E8. AG (agriculture area), IN (industrial area), UN (university area), RE (residential area), CM (commercial area). UN is the site for vertical heights samples.

All the atmospheric dustfall samples were collected under weather conditions of clear skies, calm winds, and no rainfall within one week prior to sampling; aiming to avoid the interference of precipitation scouring and wind disturbance on the integrity and representativeness of the dustfall samples. The layout of sampling sites took into account both spatial distribution characteristics and differences among functional zones. The specific arrangements for sample collection are as follows: (1) 15 road dustfall sampling sites were set along Chang’an Avenue, an east–west trunk road in Beijing; (2) 5 representative sampling sites were established in different functional areas of Beijing; (3) 6 gradient sampling sites were arranged at different vertical heights of the same building (a research building on the campus of China University of Mining and Technology, Beijing). During the period from June to July 2023, atmospheric dustfall was collected using wool brushes and aluminum foil in this study, and a total of 26 acceptable atmospheric dustfall samples were obtained. The distribution of the study area and sampling sites is shown in Figure 1. Detailed sample information is shown in Tables S1–S3.

To investigate the toxicological relationship between microplastics and atmospheric particulate matter, this study collected ambient PM_2.5_ samples on the 5th-floor platform of a research building on the campus of China University of Mining and Technology (Beijing) from September 2022 to June 2023. Several PM_2.5_ samples with relatively high pollution concentrations were selected as experimental samples. The instrument used for collecting ambient PM_2.5_ samples was a KB-120F medium-volume sampler (Gemstar, Qingdao, China) for TSP-PM_10_-PM_2.5_. The sampling flow rate was set at 100 L/min, and Ø90 mm quartz filters were used. The sampling height was 15 m above ground, and the sampling inlet was positioned 1.5 m higher than the sampling platform. During the sampling period, data on temperature (T), relative humidity (RH), and atmospheric pressure (P) were recorded using a Kestrel 5500 portable weather meter (Kestrel Meters, West Chester, PA, USA).

Polystyrene (PS) is one of the most widely used plastics and is regarded as a representative microplastic. Among them, spherical PS is uniform in both shape and size, making it a commonly used type of plastic particle in laboratory exposure studies [37]. Its inherent uniformity facilitates easy manipulation in experiments, and standard samples can also be readily obtained from commercial suppliers. The microplastic samples used in this study were standard experimental spherical PS with particle sizes of 1–2 μm and 10 μm.

According to the mass of PM_2.5_ collected on the quartz filters and microplastic samples, a maximum dose concentration of 1000 μg/mL was used, and serial dilutions were performed at an equal ratio of 5:4:3:2:1. Each sample was divided into whole samples (W) and water-soluble samples (S), with five concentration gradients prepared separately. In addition to the PM_2.5_ group, the 1–2 μm PS microplastic standard group, and the 10 μm PS microplastic standard group, two mixed groups were also set up, in which microplastic samples of different particle sizes (1–2 μm and 10 μm) were mixed with PM_2.5_ samples at an equal ratio. The sample information is shown in Table 1.

### 2.2. MP Extraction from Dustfall Samples

The density flotation method was used to separate MP particles from atmospheric dustfall [38]. Impurities and larger particles were removed from the collected atmospheric dustfall. Then, a saturated potassium formate (HCOOK) solution was prepared. Specific steps for MPs separation were as follows. (1) Atmospheric dustfall was placed in a 100 mL beaker and 70 mL of saturated HCOOK solution was added. The solution was agitated for 10 min and then allowed to settle for 24 h. (2) The supernatant was collected and vacuum filtered, and the floated particles were retained on the PTFE membrane. (3) The PTFE membrane was transferred into another 100 mL beaker, and 20 mL of 30% hydrogen peroxide (H_2_O_2_) was added to remove organic matter. After thorough stirring, the mixture was allowed to stand for 24 h to ensure a sufficient reaction between H_2_O_2_ and organic substances. (4) 60 mL of saturated HCOOK solution was added to the beaker containing H_2_O_2_, and the mixture was left to settle for 24 h. (5) The supernatant was collected and vacuum filtered again, with the floated particles retained on the PTFE membrane. If there were excessive particles on the membrane, the steps could be repeated several times to achieve purification. (6) The filter membrane was immersed in ethanol and subjected to ultrasonication to ensure that the sample on the membrane was completely eluted. (7) The filter was removed from the ethanol and rinsed until clean. When the ethanol had evaporated down to a volume of 200 μL, a drop of the ethanol was placed on a glass coverslip. After complete evaporation, leaving the MP sample adhering to the glass surface, the samples were prepared for the laser direct infrared imaging spectrometer analysis.

### 2.3. LDIR Analysis and FESEM

The laser direct infrared imaging spectrometer (LDIR) employs a quantum cascade laser (QCL) as the light source, whose output energy is more than 10,000 times that of conventional Fourier-transform infrared microscopy/infrared imaging systems [39]. LDIR can systematically detect every particle, thereby eliminating potential errors from visual selection. The instrument used in this experiment was the Agilent 8700 laser infrared imaging spectrometer (Agilent, Santa Clara, USA). When detecting microplastics using LDIR, the ATR (Attenuated Total Reflection) automatic scanning mode shall be selected, with the minimum detection particle size set to 20 μm and the maximum particle size set to 500 μm. Activate the microplastic detection module in the Clarity software, locate particles via rapid scanning at 1800 cm^−1^, automatically collect the infrared spectra of the background and particles, and perform qualitative analysis by comparison with the spectral library. Information such as particle images, particle size and area is output synchronously.

Field Emission Scanning Electron Microscopy (FESEM) uses an electron beam as the source [17]. Under an accelerating voltage, the electron beam is focused by three-stage electromagnetic lenses and scans the sample surface in a raster pattern, generating various physical signals related to sample properties (e.g., secondary electrons, backscattered electrons). These signals are then used to obtain scanning electron images that characterize the surface morphology of the sample. In this study, FESEM was used to analyze the microscopic morphology of MP particles. The MP samples were filtered onto a membrane and sprayed with aerosol gold as a conductive material and the extra high tension was set at 20 kV. The main analytical parameters are set as follows: magnification of 100–10,000×, working distance controlled within 5 mm, and observed particle size range of 20–500 μm.

### 2.4. Plasmid Scission Assay

The Plasmid Scission Assay (PSA) was conducted in accordance with the methods reported previously. (1) Based on the weight of the PM_2.5_ sample obtained from the quartz filter membrane, a corresponding amount of polystyrene (PS) microplastic was weighed and placed in a centrifuge tube. The filter membrane sample were cut into pieces and put into a 5 mL centrifuge tube, and ultrapure water added (sterilized at high temperature) to make the solution concentration reach the set maximum concentration (1000 μg/mL). The particulate matter on the filter membrane was suspended in ultrapure water by vibrating with a vortex oscillator for 20 h to obtain a bulk sample (W). Half of the whole sample was placed into a new centrifuge tube, and centrifuged (13,000 r/min; 80 min) and the supernatant taken, i.e., water-soluble sample (S). (2) The samples were prepared into 5 concentration levels, adding different proportions of sample volume and ultrapure water, then adding 2 μL of plasmid DNA and shaking horizontally on a mixer for 6 h. (3) Agarose was dissolved into a conical flask containing 1X TBE buffer solution, heated until clear, then 10 μL of ethyl bromide (EB) reagent was added when the temperature reached 78 °C. The gel solution was poured into the electrophoresis plate, placed in a proper position to form a comb to solidify the gel, and attention paid to eliminating any small bubbles above the gel solution. (4) The prepared gel in the electrophoresis tank was filled with 2 L 1× TBE buffer solution. 7 μL bromophenol blue dye was added to each sample after mixing. Using a pipette 20 μL sample solution was injected into each gel hole. After the injection was completed, 20 μL of EB reagent was added to both sides of the electrophoresis tank for color development. The electrophoresis instrument was run for 16 h. (5) After electrophoresis the gel was put into the ultraviolet light box. The ultraviolet gel imaging system observed the optical density bands of the three different forms of plasmid DNA. Using Syngene gene tool analysis software (Version 4.0) to measure its density ratio, the total percentage of relaxed and linearized plasmid DNA obtained is the damage rate of each sample. Ultrapure water was used as the blank sample in the experiment, and two parallel samples were used for each test to eliminate the influence of other factors on the experimental results.

### 2.5. Pollution Load Index

The Pollution Load Index (PLI) is an assessment method proposed in 1980 by Tomlinson et al. [40]. It is now widely applied to ecological risk assessments in the environmental field. In this study, the PLI was used to evaluate the ecological risks of microplastics in atmospheric dustfall in Beijing. The PLI of MPs at each site is related to MP concentration factors. This value does not represent the true natural background level. The calculated PLI values are only used to reflect the relative spatial differences within the study area. The PLI comprehensively considers the pollution status and correlations of MPs at local and overall scales. It also reflects the contribution of each sampling site to the overall pollution level of the study area. The assessment model was as follows [40]:

(1) $${CF}_{i}=\frac{C_{i}}{C_{0}}$$

(2) $${PLI}_{i}=\sqrt{{CF}_{i}}$$

(3) $${PLI}_{area}=\sqrt[{n}]{{PLI}_{1}\times {PLI}_{2}\times \cdots {PLI}_{n}}$$

where CF_i_ is the MP concentration factor, i.e., the ratio between the microplastic mass concentration at each location (C_i_) and the background MP concentration (C_0_). The lowest concentrations of MP detected in the dustfall samples were considered background MP concentrations. PLI at each location is related to MP concentration factors (CF_i_). The risk level was assessed based on these PLI values as reported by Xu et al. [33]: Level I (<10), II (10–20), III (20–30), and IV (>30), where Level IV indicating the highest risk level, and Level I is the lowest. Detailed contents are shown in Table 2.

### 2.6. Statistical Analysis and Quality Control

Statistical analysis of the data was conducted using GraphPad Prism 10. One-way analysis of variance (ANOVA) and linear trend test were used to analyze and evaluate the correlation between experimental doses and DNA damage rate. The t-test was used to assess the correlation between microplastic particle sizes and DNA damage rate across different sample types. Statistical significance was established at a threshold of *p* < 0.05.

To minimize external contamination as much as possible, preventive measures were taken. Throughout the entire experiment, pure cotton lab coats, masks, and nitrile gloves were worn. During outdoor sampling, again, pure cotton clothing, masks and nitrile gloves were worn. Plastic consumables and tools were avoided, and consumables and tools were covered in metal foil prevent contamination when not in use. The reagents and solutions used in the experiments were filtered through a membrane before use. All glassware (beakers, measuring cylinders, filtering devices, etc.) and stainless-steel tools were rinsed with distilled water and dried by treating with alcohol before the experiments. In the experiments, blank filter membranes were used as negative controls, undergoing the same experimental procedures as the experimental groups, and values obtained treated as the background value in the subsequent calculations and analysis.

## 3. Results and Discussion

### 3.1. Abundance of Microplastics

**Figure 2:**
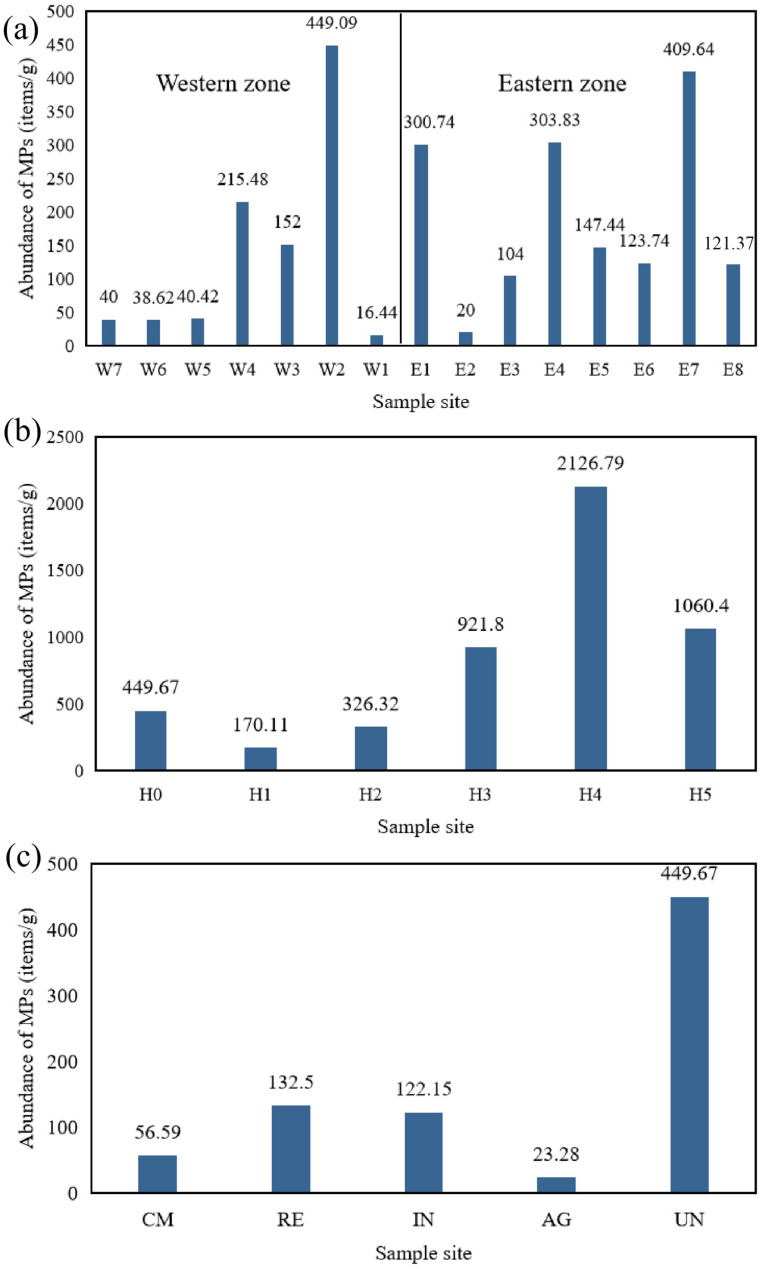
Abundance of microplastics at different sampling sites in Beijing. (**a**) Abundance of microplastics in road dustfall in Beijing. (**b**) Abundance of microplastics in atmospheric dustfall at vertical heights in Beijing. (**c**) Microplastic abundance in atmospheric precipitation at sampling sites in different functional areas of Beijing. AG (agricultural area), IN (industrial area), UN (university area), CM (commercial area), and RE (residential area).

In this study, the abundance of microplastics refers to the number of MPs per gram of dustfall or per cubic metre of air. The abundance of microplastics in road dustfall at sampling sites in Beijing are shown in Figure 2a. Among the 15 sampling sites, the abundance of microplastics ranged from 16.44 to 449.09 items/g, with an average value of 165.52 items/g. The highest microplastic abundance was observed at sampling site W2 (449.09 items/g), followed by E7 (409.64 items/g), E4 (303.83 items/g), E1 (300.74 items/g), W4 (215.48 items/g), W3 (152 items/g), E5 (147.44 items/g), E6 (123.74 items/g), E8 (121.37 items/g), E3 (104 items/g), W5 (40.42 items/g), W7 (40 items/g), W6 (38.62 items/g), E2 (20 items/g), and W1 (16.44 items/g), in a descending order.

Overall, the 15 sampling sites were divided into eastern and western zones along the central axis of Beijing. The spatial distribution of microplastics differed significantly between the eastern zone (E1–E8) and the western zone (W1–W7). The average abundance in the eastern zones was 191.35 items/g, which was higher than the overall average, while that in the western zone was 136.01 items/g. The average microplastic abundance in the eastern zone was approximately 1.4 times that in the western zone. The eastern zone includes Chaoyang District, the most populous district in Beijing, and Tongzhou District, the sub-center of Beijing. It has a denser road network and more developed transportation, which may explain why the abundance of microplastics in the eastern zone is higher than that in the western zone. In a study of microplastics in roadside soils along Chang’an Avenue and its extension, Zhang et al. found that the abundance of microplastics in the eastern zone was higher than that in the western zone [41]. When investigating microplastics in atmospheric dustfall along the north–south central axis of Beijing, Liu et al. observed that microplastic abundance was higher in the southern zone than in the northern zone [39]. These findings are generally consistent with the spatial distribution pattern of population density in Beijing, suggesting that urban microplastic abundance is correlated with population density; regions with higher population density tend to have higher microplastic abundance. It is noted that the prevailing wind direction in Beijing is NNW, so approximately at right angles to the sample W–E axis.

The abundance of microplastics in atmospheric dustfall at sampling sites of different heights in Beijing are shown in Figure 2b. Among the six sampling highs, the abundance of microplastics ranged from 170.11 to 2126.79 items/g, with an average value of 842.51 items/g. The highest microplastic abundance was found at the 4th floor sampling site (2126.79 items/g, height: 13.6 m), followed by the 5th floor (1060.40 items/g), 3rd floor (921.80 items/g), ground floor (0th floor, 449.67 items/g), 2nd floor (326.32 items/g), and 1st floor (170.11 items/g). Compared with lower floors, higher floors exhibit relatively higher microplastic abundances. Studies have shown that human activities and meteorological factors (airflow disturbance) are key factors affecting the abundance and spatiotemporal distribution of microplastics [42]. Since the sampling sites on different floors are located on windowsills near ventilation openings, it is inferred that microplastics generated by indoor human activities may be the primary source.

Different functional zones can be associated with different abundances of microplastics in the dustfall (Figure 2c). Among the five functional areas, the abundance of microplastics ranged from 23.28 to 449.67 items/g, with an average value of 156.84 items/g. The highest microplastic abundance was observed at the sampling site in the university area (site UN: 449.67 items/g), followed by the residential area (site RE: 132.50 items/g), industrial area (site IN: 122.15 items/g), commercial area (site CM: 56.59 items/g), and agricultural area (site AG: 23.28 items/g). University, residential, commercial, and industrial areas have higher pedestrian flows than the agricultural area, which may result in relatively higher microplastic abundance. Studies have suggested that agricultural land contributes the least to total environmental microplastics compared with urban roads [43]. Brahney et al. found that in the western United States, microplastics accounts for only 5% of atmospheric particles at the agriculture sites, whereas microplastics contribute 84% at the road sites [44]. These findings are consistent with the results of the present study.

### 3.2. Oxidative Damage Capacity of PM_2.5_ and Microplastic Samples at Different Dose Concentrations

After density separation, the mass of microplastics obtained from the atmospheric dustfall samples was limited and insufficient for PSA analysis. In addition, the solvents used during the extraction process may interfere with the detection of toxic effects. Therefore, we selected plastic particles (PS), which are commonly used in laboratory exposure studies, as representative microplastics to directly conduct the preliminary oxidative damage experiments.

The oxidative capacity of the samples in Table 1 was evaluated by the plasmid DNA damage method, and the DNA damage rates are shown in Table 3. Among the observed samples (a–e), the DNA damage rate of both whole samples and water-soluble samples increased with the increase in experimental dose. Overall, at the same dose concentration, the DNA damage rate of all whole samples is higher than that of water-soluble samples.

**Figure 3:**
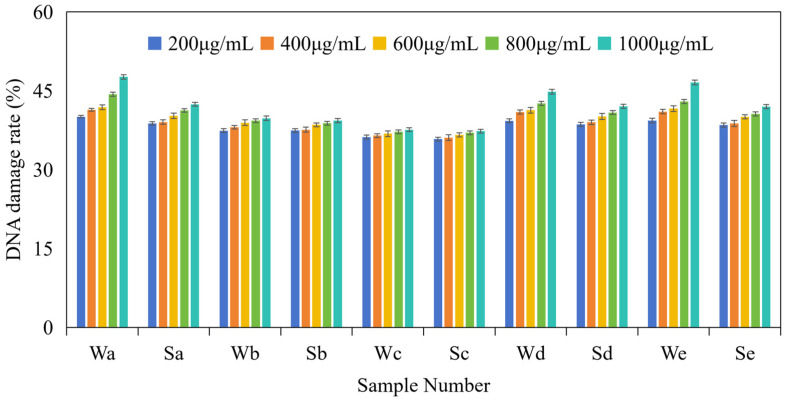
Histogram of DNA damage rate of PM_2.5_ and microplastic samples at different dose concentrations. Sample numbers correspond to Table 1 and Table 3.

At the dose concentrations ranging from 200 to 1000 μg/mL, the DNA damage rates of PM_2.5_ samples and microplastic standard samples were between 35.81% and 47.64%. Among them, the DNA damage rate of the whole samples ranged from 36.22% to 47.64%, while that of the water-soluble samples ranged from 35.81% to 42.42%. A plot of the DNA damage rates of PM_2.5_ samples and microplastic samples at doses of 200–1000 μg/mL, as a histogram, is shown in Figure 3. From this histogram, it is observed that there is a positive correlation between the DNA damage rate of PM_2.5_ samples and microplastic samples and the experimental doses (Significance tests are shown in Table S4). The DNA damage rate of PM_2.5_ samples and microplastic samples increases with the concentration of experimental doses, and the maximum damage rate of the samples usually occurs at the highest doses. This result is consistent with the results of Feng et al., which suggested that the oxidative capacity of PM_2.5_ was positively correlated with its dose concentration [31]. In addition, some studies have also shown that microplastics show a dose–response relationship; when the concentration of microplastics is higher, the genetic toxicity is stronger [45,46,47,48]. Therefore, the research findings preliminarily indicate that higher particulate matter concentrations cause greater potential harm to human health.

There are two main mechanisms by which particulate matter damages DNA; mechanical damage and oxidative damage [31]. Mechanical damage refers to DNA breakage caused by direct contact, collision and abrasion between particles and DNA, while oxidative damage refers to excessive free radicals or reactive oxygen species produced by particles after being dissolved in water, breaking the redox balance and leading to DNA unspinning and breakage. Atmospheric dustfall samples and PM_2.5_ samples may carry toxic substances in the environment, which can cause DNA damage. Microplastics in environmental samples may contain substances such as plasticizers, stabilizers, and antioxidants, which can also cause DNA damage. Water-soluble fraction of the samples do not contain solid particles, and the DNA damage caused is mainly oxidative damage [49]. Comparing the experimental results, it is found that, at the same concentration, the DNA damage rate of water-soluble fraction samples is marginally lower, but relatively similar, to the whole sample DNA damage. According to some studies, it has been shown that microplastics can cause DNA damage through direct or indirect interactions with genetic material, such as by the formation of reactive oxygen species [46,50].

### 3.3. Oxidative Capacity and Dose–Response Relationship of Microplastics and PM_2.5_ Mixtures with Different Particle Size

**Figure 4:**
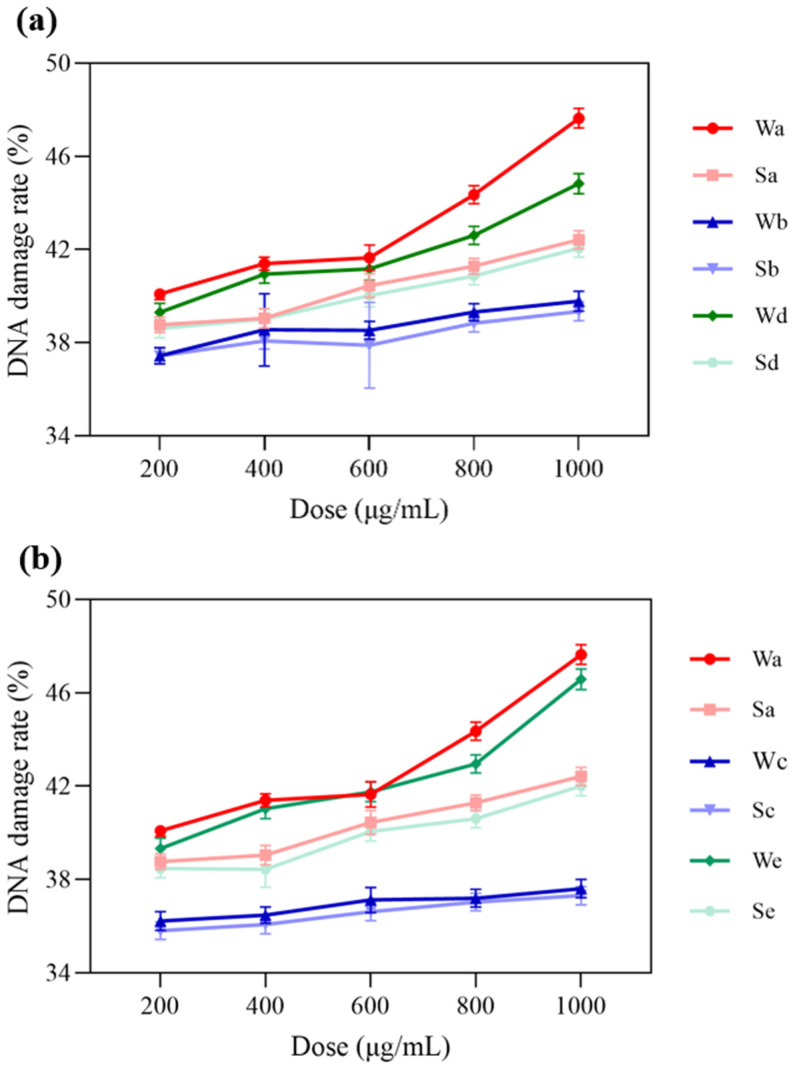
Effects of particle size (1–2 μm and 10 μm) and dose on DNA damage rates induced by PM_2.5_ and microplastics. (**a**) Relationships between the DNA damage rate of PM_2.5_ and microplastics samples with a 1–2 μm particle size at different doses. (**b**) Relationships between the DNA damage rate of PM_2.5_ and microplastics samples with a 10 μm particle size at different doses.

The DNA damage rates of microplastic samples with particle sizes of 1–2 μm and 10 μm and PM_2.5_ samples at different dose concentrations are shown in Figure 4. In Table 3 the DNA damage rate of 1–2 μm microplastic samples ranged from 37.43% to 39.78%, while that of 10 μm microplastic samples ranged from 35.81% to 37.61%. Through statistical analysis, we examined the particle size effect of microplastic samples of different sizes (Tables S5 and S6). We preliminarily found that the microplastic samples with smaller particle sizes exhibited slightly higher DNA damage rates. Indicating that the oxidative damage capacity of these microplastics may be affected by particle size, possibly as a function of their increased reactive surface areas. In addition, it is seen that for 1–2 μm or 10 μm microplastic samples, the DNA damage rate after mixing with PM_2.5_ samples is between the DNA damage rates of PM_2.5_ samples and microplastic samples.

### 3.4. Pollution Load Index of Microplastics in Atmospheric Dustfall in Beijing

**Figure 5:**
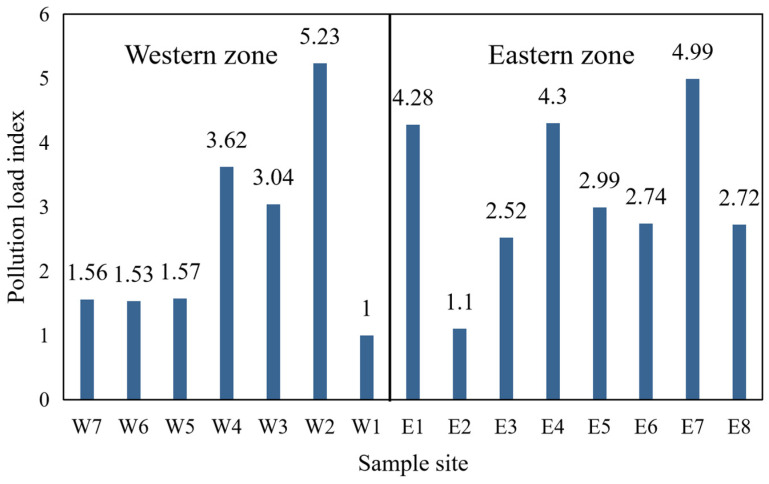
Pollution load index bar chart of microplastics from road dustfall in Beijing. Sample site corresponding to Table S1.

The PLI of sampling sites along Chang’an Avenue was calculated (as shown in Figure 5), with an average of 2.88. For sampling sites in the western zone of Beijing, the PLI ranged from 1.00 to 5.23, with the highest value observed at W2 and the lowest at W1. The overall PLI for the entire western zone was 2.51, indicating light pollution. For sampling sites in the eastern zone, the PLI ranged from 1.1 to 4.99, with the highest value at E7 and the lowest at E2. The overall PLI for the entire eastern zone was 3.21, it is higher than that in the western zone.

**Figure 6:**
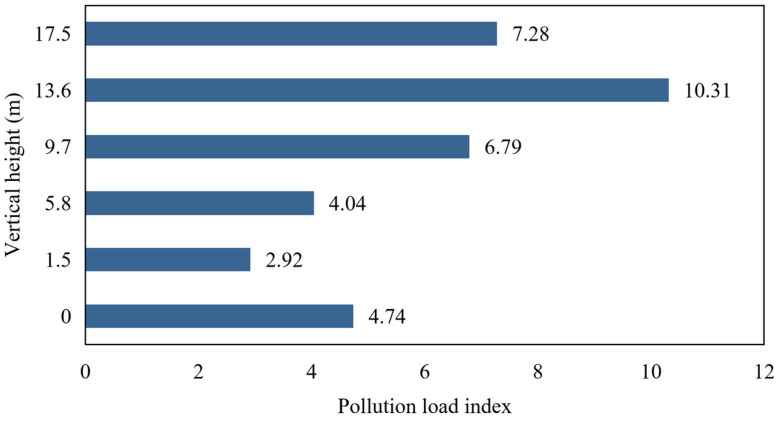
Pollution load index bar chart of microplastics in atmospheric dustfall at vertical heights in Beijing.

The PLI of microplastics in atmospheric dustfall at different vertical heights in Beijing is shown in Figure 6. The highest microplastic PLI was found on the 4th floor (PLI_H4_ = 3.54), and the lowest at the 1st floor (PLI_H1_ = 1). The PLI values were 1.63 for the ground floor, 1.39 for the 2nd floor, 2.33 for the 3rd floor, and 2.5 for the 5th floor. All samples collected from different floors exhibited light pollution. Overall, the PLI followed the order: 4th floor > 5th floor > 3rd floor > ground floor > 2nd floor > 1st floor. Higher floors were subject to a relatively higher degree of contamination, which deserves special attention.

Table 4 shows the Pollution Load Index (PLI) of microplastics in atmospheric dustfall across different functional areas in Beijing. The highest PLI of microplastics was found in the university area (UN: 4.39), while the lowest was in the agricultural area (AG: 1.00). The PLI values were 2.39 for the residential area, 1.56 for the commercial area, and 2.29 for the industrial area. All five functional areas were classified as light pollution. Overall, the PLI followed the order: university area > residential area > industrial area > commercial area > agricultural area. The university area exhibited the highest pollution level, which may be attributed to high population mobility and infrequent cleaning, favoring the accumulation of microplastics. Although the commercial area also had high population mobility, its pollution level was lower than that of the university area. This may be due to frequent cleaning by shopping mall staff, which disrupts microplastic accumulation. Another possible reason is that the sampling sites were close to multiple building entrances and exits, where airflow generated by pedestrian movement can cause secondary transport of microplastics. The agricultural area is located on the urban fringe with a sparse population and relatively low plastic usage, resulting in the lowest pollution level.

### 3.5. Limitations and Future Perspectives

1. This study used an in vitro PSA to assess microplastic-induced oxidative damage, which only indicates microplastics’ potential DNA damage risk and cannot reflect real in vivo toxicity. Limited by high purification difficulty and impurity interference, only commercial polystyrene microplastics were tested instead of real environmental samples, failing to show their actual toxicological traits. Future work can build live-cell biological systems, optimize atmospheric microplastic extraction, and test real environmental and multi-type microplastics for accurate risk assessment.
2. Due to the lack of a unified atmospheric microplastic background value in Beijing, the minimum sampling site concentration was used to calculate the Pollution Load Index, which only reflects relative pollution differences, and absolute pollution levels need verification. Future long-term large-scale regional monitoring is needed to improve the PLI system and enhance assessment accuracy.

## 4. Conclusions

1. The plasmid scission assay on PM_2.5_ in the environmental atmosphere of Beijing showed a clear dose–response relationship. Similarly, the DNA damage rate of microplastic samples was increasing with experimental dose, that is, the higher the dose concentration, the stronger the oxidative damage. Therefore, high concentrations of particulate matter may have potential implications for human health, which requires further in-depth research to confirm.
2. For both 1–2 μm and 10 μm microplastic samples, the DNA damage rate of their 1:1 mixture with PM_2.5_ samples showed a clear dose–response relationship, indicating that atmospheric microplastic samples can induce toxicological effects.
3. There are some differences in oxidative capacity of microplastics with different particle sizes, and the DNA damage rate of 1–2 μm is slightly higher than that of microplastic samples of 10 μm. This suggests that the oxidative potential of the finer microplastics may cause more obvious damage to plasmid DNA.
4. According to the PLI calculation results, the pollution level of microplastics in atmospheric dustfall in Beijing shows a clear spatial variation, with the eastern zone being generally higher than the western zone. A comparison among different functional areas showed a trend that university area > residential area > industrial area > commercial area > agricultural area. Among samples collected at different vertical heights, higher floors exhibit a higher pollution level than lower floors, which deserves special attention.

## Figures and Tables

**Table 1:** Sample information of PM_2.5_ and MPs.

Sample Number	Sample Information
a	PM_2.5_ sample
b	PS microplastic standard sample with particle size of 1–2 μm
c	PS microplastic standard sample with particle size of 10 μm
d	PS microplastic standard samples with particle size of 1–2 μm were mixed with PM_2.5_ samples in equal ratio 1:1
e	PS microplastic standard samples with particle size of 10 μm were mixed with PM_2.5_ samples in equal ratio 1:1

**Table 2:** Criteria for assessment of pollution load index (PLI).

**PLI**	<10	10–20	20–30	>30
**Risk Category**	I	II	III	IV
**Pollution Level**	Light pollution	Moderate pollution	Severe pollution	Extreme pollution

**Table 3:** DNA damage rate (%) of PM_2.5_ and microplastic standard samples at different dose concentrations. W: bulk samples; S: water-soluble samples.

Sample Number	Sample Type	Experimental Dosage (μg/mL)				
		200	400	600	800	1000
a	W	40.08	41.39	41.88	44.35	47.64
	S	38.77	39.05	40.25	41.28	42.42
b	W	37.43	38.07	38.93	39.31	39.78
	S	37.44	37.61	38.53	38.83	39.34
c	W	36.22	36.47	36.85	37.20	37.61
	S	35.81	36.08	36.62	37.03	37.31
d	W	39.30	40.94	41.31	42.61	44.83
	S	38.60	39.01	40.13	40.86	42.06
e	W	39.33	41.04	41.62	42.95	46.58
	S	38.48	38.78	40.06	40.60	42.00

**Table 4:** Microplastic pollution load index and pollution level of dustfall in different functional areas of Beijing.

Functional Areas	PLI	Pollution Level
Commercial area	1.56	Light pollution
Residential area	2.39	Light pollution
Industrial area	2.29	Light pollution
Agricultural area	1.00	Light pollution
University area	4.39	Light pollution

## Data Availability

All the data presented in this paper is available upon request. Please contact the corresponding author (Longyi Shao: shaol@cumtb.edu.cn).

## Supplementary Materials

The following supporting information can be downloaded at: https://www.mdpi.com/article/10.3390/toxics14040312/s1, Table S1: Information on road dustfall sampling sites; Table S2: Information on sampling sites in different functional areas; Table S3: Information on sampling sites at different vertical heights of the same building; Table S4: The significance test of the correlation between experimental dosage and DNA damage rate (%); Table S5: The significance test of the correlation between different particle sizes microplastics (1–2 μm and 10 μm) and DNA damage rate (%). (Wb vs. Wc); Table S6: The significance test of the correlation between different particle sizes microplastics (1–2 μm and 10 μm) and DNA damage rate (%). (Sb vs. Sc).

## References

[1] Cottom J.W., Cook E., Velis C.A. (2024). A local-to-global emissions inventory of macroplastic pollution. Nature.

[2] Stoett P., Scrich V.M., Elliff C.I., Andrade M.M., Grilli N.d.M., Turra A. (2024). Global plastic pollution, sustainable development, and plastic justice. World Dev..

[3] Thompson R.C., Courtene-Jones W., Boucher J., Pahl S., Raubenheimer K., Koelmans A.A. (2024). Twenty years of microplastic pollution research—What have we learned?. Science.

[4] Thompson R.C., Olsen Y., Mitchell R.P., Davis A., Rowland S.J., John A.W.G., McGonigle D., Russell A.E. (2004). Lost at sea: Where is all the plastic?. Science.

[5] Hartmann N.B., Hüffer T., Thompson R.C., Hassellöv M., Verschoor A., Daugaard A.E., Rist S., Karlsson T., Brennholt N., Cole M. (2019). Are we speaking the same language? Recommendations for a definition and categorization framework for plastic debris. Environ. Sci. Technol..

[6] Yan M., Nie H., Xu K., He Y., Hu Y., Huang Y., Wang J. (2019). Microplastic abundance, distribution and composition in the Pearl River along Guangzhou city and Pearl River Estuary, China. Chemosphere.

[7] Galafassi S., Nizzetto L., Volta P. (2019). Plastic sources: A survey across scientific and grey literature for their inventory and relative contribution to microplastics pollution in natural environments, with an emphasis on surface water. Sci. Total Environ..

[8] Salawu O.A., Olivares C.I., Adeleye A.S. (2024). Adsorption of PFAS onto secondary microplastics: A mechanistic study. J. Hazard. Mater..

[9] Evangeliou N., Grythe H., Klimont Z., Heyes C., Eckhardt S., Lopez-Aparicio S., Stohl A. (2020). Atmospheric transport is a major pathway of microplastics to remote regions. Nat. Commun..

[10] Zhang Y., Gao T., Kang S., Sillanpää M. (2019). Importance of atmospheric transport for microplastics deposited in remote areas. Environ. Pollut..

[11] Ambrosini R., Azzoni R.S., Pittino F., Diolaiuti G., Franzetti A., Parolini M. (2019). First evidence of microplastic contamination in the supraglacial debris of an Alpine Glacier. Environ. Pollut..

[12] Allen S., Allen D., Phoenix V.R., Le Roux G., Durántez Jiménez P., Simonneau A., Binet S., Galop D. (2019). Atmospheric transport and deposition of microplastics in a remote mountain catchment. Nat. Geosci..

[13] Bergmann M., Mützel S., Primpke S., Tekman M.B., Trachsel J., Gerdts G. (2019). White and wonderful? Microplastics prevail in snow from the Alps to the Arctic. Sci. Adv..

[14] Zhang N., Zhang C., Qin Y., Wang J., Ge X., Li H., Dai Y., Aruffo E. (2024). A review of atmospheric microplastics: Sources, characteristics, and detection method. Curr. Pollut. Rep..

[15] Wright S.L., Ulke J., Font A., Chan K.L.A., Kelly F.J. (2020). Atmospheric microplastic deposition in an urban environment and an evaluation of transport. Environ. Int..

[16] Huang L., Zhang S., Li L., Zhang S., Wang J., Liu X., Zhang W. (2023). Research progress on microplastics pollution in Polar Oceans. Polar Sci..

[17] Liu P., Shao L., Zhang Y., Silvonen V., Oswin H., Cao Y., Guo Z., Ma X., Morawska L. (2024). Atmospheric microplastic deposition associated with GDP and population growth: Insights from megacities in northern China. J. Hazard. Mater..

[18] He G., Xie H., Tan B., Chen M., Wu Z., Dai Z., Sun R., He L., Li C. (2025). Effects of microplastics and heavy metal stress on the growth and physiological characteristics of pioneer plant Avicennia marina. Mar. Pollut. Bull..

[19] Arias A.H., Alvarez G., Pozo K., Pribylova P., Klanova J., Rodríguez Pirani L.S., Picone A.L., Alvarez M., Tombesi N. (2023). Beached microplastics at the Bahia Blanca estuary (Argentina): Plastic pellets as potential vectors of environmental pollution by POPs. Mar. Pollut. Bull..

[20] Hou G., Zhao X., Zhao T., Wu X., Pu S., Tang Z., Wu F. (2023). The adsorption of PAHs on microplastics and desorption in the simulated human digestive system. Chem. Eng. J..

[21] Cheung C.K.H., Not C. (2023). Impacts of extreme weather events on microplastic distribution in coastal environments. Sci. Total Environ..

[22] Sharma V.K., Ma X., Lichtfouse E., Robert D. (2023). Nanoplastics are potentially more dangerous than microplastics. Environ. Chem. Lett..

[23] Wang Y.-L., Lin Y.-C., Liu W.-C., Lee Y.-H., Chiu H.-W. (2025). Air pollution and its impacts on health: Focus on microplastics and nanoplastics. Ecotoxicol. Environ. Saf..

[24] Bucur R.M., Radulescu C., Dulama I.D., Stirbescu R.M., Bucurica I.A., Banica A.L., Stanescu S.G. (2025). Potential health risk of microplastic exposures from skin-cleansing products. Toxics.

[25] Kopatz V., Wen K., Kovács T., Keimowitz A.S., Pichler V., Widder J., Vethaak A.D., Hollóczki O., Kenner L. (2023). Micro- and nanoplastics breach the blood–brain barrier (BBB): Biomolecular corona’s role revealed. Nanomaterials.

[26] Yuan W., Liu X., Wang W., Di M., Wang J. (2019). Microplastic abundance, distribution and composition in water, sediments, and wild fish from Poyang lake, China. Ecotoxicol. Environ. Saf..

[27] Zhu W., Zhao N., Liu W., Guo R., Jin H. (2023). Occurrence of microplastics in Antarctic fishes: Abundance, size, shape, and polymer composition. Sci. Total Environ..

[28] Rafa N., Ahmed B., Zohora F., Bakya J., Ahmed S., Ahmed S.F., Mofijur M., Chowdhury A.A., Almomani F. (2024). Microplastics as carriers of toxic pollutants: Source, transport, and toxicological effects. Environ. Pollut..

[29] Shao L., Hu Y., Shen R., Schäfer K., Wang J., Wang J., Schnelle-Kreis J., Zimmermann R., BéruBé K., Suppan P. (2017). Seasonal variation of particle-induced oxidative potential of airborne particulate matter in Beijing. Sci. Total Environ..

[30] Xue X., Yang S., Fan S., Cao Y., Wang W., Shao L. (2026). Exposure toxicity of dust storm particles based on plasmid scission assay: An example from Beijing. Atmosphere.

[31] Feng X., Shao L., Jones T., Li Y., Cao Y., Zhang M., Ge S., Yang C.-X., Lu J., BéruBé K. (2022). Oxidative potential and water-soluble heavy metals of size-segregated airborne particles in haze and non-haze episodes: Impact of the “Comprehensive action plan” in China. Sci. Total Environ..

[32] Lawson M.J., Prytherch Z.C., Jones T.P., Adams R.A., BéruBé K.A. (2020). Iron-rich magnetic coal fly ash particles induce apoptosis in human bronchial cells. Appl. Sci..

[33] Xu P., Peng G., Su L., Gao Y., Gao L., Li D. (2018). Microplastic risk assessment in surface waters: A case study in the Changjiang estuary, China. Mar. Pollut. Bull..

[34] Joshi C., Phyllei S.W.E., Bhatt S., Chatterjee S. (2025). Microplastic surge in the Ariyankuppam River, Puducherry, India: A study on abundance, characterization, and pollution load index. J. Contam. Hydrol..

[35] Cole M., Lindeque P., Halsband C., Galloway T.S. (2011). Microplastics as contaminants in the marine environment: A review. Mar. Pollut. Bull..

[36] Ranjani M., Veerasingam S., Venkatachalapathy R., Mugilarasan M., Bagaev A., Mukhanov V., Vethamony P. (2021). Assessment of potential ecological risk of microplastics in the coastal sediments of India: A meta-analysis. Mar. Pollut. Bull..

[37] Hamm T., Lenz M. (2021). Negative impacts of realistic doses of spherical and irregular microplastics emerged late during a 42 weeks-long exposure experiment with blue mussels. Sci. Total Environ..

[38] Shao L., Li Y., Jones T., Santosh M., Liu P., Zhang M., Xu L., Li W., Lu J., Yang C.-X. (2022). Airborne microplastics: A review of current perspectives and environmental implications. J. Clean. Prod..

[39] Liu P., Shao L., Li Y., Jones T., Cao Y., Yang C.-X., Zhang M., Santosh M., Feng X., BéruBé K. (2022). Microplastic atmospheric dustfall pollution in urban environment: Evidence from the types, distribution, and probable sources in Beijing, China. Sci. Total Environ..

[40] Tomlinson D.L., Wilson J.G., Harris C.R., Jeffrey D.W. (1980). Problems in the assessment of heavy-metal levels in estuaries and the formation of a pollution index. Helgoländer Meeresunters..

[41] Zhang M., Liu L., Xu D., Zhang B., Li J., Gao B. (2022). Small-sized microplastics (<500 Μm) in roadside soils of Beijing, China: Accumulation, stability, and human exposure risk. Environ. Pollut..

[42] Zhang Q., Zhao Y., Du F., Cai H., Wang G., Shi H. (2020). Microplastic fallout in different indoor environments. Environ. Sci. Technol..

[43] Rostami S., Talaie M.R., Talaiekhozani A., Sillanpää M. (2021). Evaluation of the available strategies to control the emission of microplastics into the aquatic environment. Environ. Sci. Pollut. Res..

[44] Brahney J., Mahowald N., Prank M., Cornwell G., Klimont Z., Matsui H., Prather K.A. (2021). Constraining the atmospheric limb of the plastic cycle. Proc. Natl. Acad. Sci. USA.

[45] Hu M., Palić D. (2020). Role of microRNAs in regulation of DNA damage in monocytes exposed to polystyrene and TiO_2_ nanoparticles. Toxicol. Rep..

[46] Malinowska K., Bukowska B., Piwoński I., Foksiński M., Kisielewska A., Zarakowska E., Gackowski D., Sicińska P. (2022). Polystyrene nanoparticles: The mechanism of their genotoxicity in human peripheral blood mononuclear cells. Nanotoxicology.

[47] Møller P., Roursgaard M. (2023). Exposure to nanoplastic particles and DNA damage in mammalian cells. Mutat. Res.-Rev. Mutat. Res..

[48] Shi X., Wang X., Huang R., Tang C., Hu C., Ning P., Wang F. (2022). Cytotoxicity and genotoxicity of polystyrene micro- and nanoplastics with different size and surface modification in A549 cells. Int. J. Nanomed..

[49] Alnasser S.M. (2025). Revisiting the approaches to DNA damage detection in genetic toxicology: Insights and regulatory implications. BioData Min..

[50] Sarma D.K., Dubey R., Samarth R.M., Shubham S., Chowdhury P., Kumawat M., Verma V., Tiwari R.R., Kumar M. (2022). The biological effects of polystyrene nanoplastics on human peripheral blood lymphocytes. Nanomaterials.

