# Supplementary material for: A Toxicological Assessment of Airborne Microplastics in Beijing
Source: Toxics. 2026 Apr 7;14(4):312. doi: 10.3390/toxics14040312 (PMC13120077; doi:10.3390/toxics14040312)
Supplement: Supplementary file 1 [file toxics-14-00312-s001.zip › toxics-4196972-supplementary.pdf]

Supplementary Information

A toxicological assessment of airborne microplastics in Beijing

Susu Fan [1], Ziyu Guo [1], Longyi Shao [1*], Pengju Liu [2], Tim Jones[3], Yaxin Cao [1], Wen-jing Deng [4], Hong Li [5], Kelly BéruBé [6]

1 State Key Laboratory of Coal Resources and Safe Mining, School of Geoscience and Survey Engineering, China University of Mining and Technology, Beijing 100083, China

2 State Key Laboratory of Regional Environment and Sustainability, School of Environment, Tsinghua University, Beijing 100084, China

3 School of Earth and Ocean Sciences, Cardiff University, Main Building, Park Place, Cardiff, CF10 3AT

4 Department of Science and Environmental Studies, The Education University of Hong Kong, Tai Po, N.T., Hong Kong, China

5 State Key Laboratory of Environmental Criteria and Risk Assessment, Chinese Research Academy of Environmental Sciences, Beijing 100012, China

6 School of Biosciences, Cardiff University, the Sir Martin Evans Building, Museum Avenue, Cardiff, CF10 3AX

* Correspondence: ShaoL@cumtb.edu.cn

Table S1. Information on road dustfall sampling sites.

Sampling Site ID	Sampling Date	Longitude (°)	Latitude (°)
**E1**	June 9, 2023	116.42115	39.91424
**E2**	June 9, 2023	116.45717	39.91399
**E3**	June 9, 2023	116.49106	39.91385
**E4**	June 9, 2023	116.52545	39.91399
**E5**	June 25, 2023	116.56048	39.91478
**E6**	June 25, 2023	116.59658	39.91470
**E7**	June 25, 2023	116.63218	39.91311
**E8**	June 25, 2023	116.67036	39.91435
**W1**	June 9, 2023	116.38603	39.91306
**W2**	June 9, 2023	116.35117	39.91272
**W3**	June 10, 2023	116.29683	39.91363
**W4**	June 10, 2023	116.27599	39.91364
**W5**	June 24, 2023	116.23998	39.91305
**W6**	June 24, 2023	116.20395	39.91319
**W7**	June 24, 2023	116.16583	39.91425

Table S2. Information on sampling sites in different functional areas.

Sampling Site ID	Functional Area	Sampling Date	Longitude (°)	Latitude (°)
**AG**	Agricultural area	June 11, 2023	116.50956	39.71337
**IN**	Industrial area	June 11, 2023	116.52388	39.73485
**CM**	Commercial area	July 11, 2023	116.39989	40.00756
**RE**	Residential area	July 11, 2023	116.41076	40.00662
**UN**	University area	June 11, 2023	116.35792	40.00325

Table S3. Information on sampling sites at different vertical heights of the same building.

Sampling Site ID	Floor	Height above ground	Longitude (°)	Latitude (°)
**H0**	Ground	0	116.35792	40.00325
**H1**	1	1.5	116.35792	40.00325
**H2**	2	5.8	116.35792	40.00325
**H3**	3	9.7	116.35792	40.00325
**H4**	4	13.6	116.35792	40.00325
**H5**	5	17.5	116.35792	40.00325

Table S4. The significance test of the correlation between experimental dosage and DNA damage rate (%).

Sample Number	Experimental dosage (μg/mL)	DNA damage rate (%)	Variance	Standard Deviation	Notes (ANOVA)
**Wa**	**200**	40.08	0.0553	0.2352	F=375.44，P<0.001；No outliers, extremely significant dose-effect relationship.
	**400**	41.39	0.0787	0.2805	
	**600**	41.88	0.3022	0.5498	
	**800**	44.35	0.1519	0.3897	
	**1000**	47.64	0.1767	0.4204	
**Sa**	**200**	38.77	0.1129	0.336	F=89.62, P<0.001; No outliers, extremely significant dose-effect relationship
	**400**	39.05	0.1664	0.408	
	**600**	40.25	0.2529	0.5029	
	**800**	41.28	0.1123	0.3351	
	**1000**	42.42	0.1459	0.382	
**Wb**	**200**	37.43	0.1213	0.348	F=20.43, P<0.001; No outliers, extremely significant dose-effect relationship
	**400**	38.07	0.1183	0.344	
	**600**	38.93	0.1444	0.38	
	**800**	39.31	0.1333	0.365	
	**1000**	39.78	0.1813	0.426	
**Sb**	**200**	37.44	0.1291	0.359	F=1.94, P>0.05; No significant difference between groups, non-significant trend
	**400**	37.61	2.4281	1.558	
	**600**	38.53	0.1444	0.38	
	**800**	38.83	0.1321	0.363	
	**1000**	39.34	0.1552	0.394	
**Wc**	**200**	36.22	0.1636	0.4045	F=22.18, P<0.001; Extremely significant
	**400**	36.47	0.1183	0.344	

	**600**	36.85	2.0325	1.4257	difference between groups, extremely significant trend
	**800**	37.2	0.1444	0.38	
	**1000**	37.61	0.1573	0.3966	
	**200**	35.81	0.1417	0.376	
	**400**	36.08	3.006	1.734	
**Sc**	**600**	36.62	0.1543	0.393	F=12.06, P<0.001; Extremely significant difference between groups, significant trend
	**800**	37.03	0.1371	0.37	
	**1000**	37.31	0.1573	0.397	
	**200**	39.3	0.1519	0.39	
	**400**	40.94	0.1501	0.387	
**Wd**	**600**	41.31	0.2299	0.479	F=138.80, P<0.001; No outliers, excellent dose-effect relationship
	**800**	42.61	0.1501	0.387	
	**1000**	44.83	0.1861	0.431	
	**200**	38.6	0.1519	0.39	
	**400**	39.01	0.1813	0.426	
**Sd**	**600**	40.13	0.2353	0.485	F=111.20, P<0.001; No outliers, good dose-effect relationship
	**800**	40.86	0.1351	0.368	
	**1000**	42.06	0.1492	0.386	
	**200**	39.33	0.1911	0.437	
	**400**	41.04	0.1872	0.433	
**We**	**600**	41.62	0.1753	0.419	F=491.20, P<0.001; No outliers, excellent dose-effect relationship
	**800**	42.95	0.1519	0.39	
	**1000**	46.58	0.1929	0.439	
	**200**	38.48	0.1648	0.406	
	**400**	38.78	0.5932	0.77	
**Se**	**600**	40.06	0.1708	0.413	F=49.91, P<0.001; Extremely significant trend
	**800**	40.6	0.1444	0.38	
	**1000**	42	0.1609	0.401	

Table S5. The significance test of the correlation between different particle sizes microplastics (1-2 μm and 10μm) and DNA damage rate (%). (Wb vs Wc)

Experimental dosage (μg/mL)	t-value	P-value	Significance marker
**200**	4.27	0.013	*
**400**	2.21	0.08	
**600**	4.12	0.015	*
**800**	8.23	0.001	**
**1000**	7.65	0.002	**

Table S6. The significance test of the correlation between different particle sizes microplastics (1-2 μm and 10μm) and DNA damage rate (%).(Sb vs Sc).

Experimental dosage (μg/mL)	t-value	P-value	Significance marker
**200**	7.89	0.001	**

**400**	9.12	0.001	**
**600**	1.23	0.27	
**800**	8.56	0.001	**
**1000**	7.34	0.002	**
